# Cognitive Impairment After Stroke

**DOI:** 10.7759/cureus.335

**Published:** 2015-09-29

**Authors:** Pushpendra Nath Renjen, Charu Gauba, Dinesh Chaudhari

**Affiliations:** 1 Neurosciences, Indraprastha Apollo Hospital; 2 Neurosciences, Indraprastha Apollo Hospitals; 3 Internal Medicine / Neurosciences, Indraprastha Apollo Hospital

**Keywords:** cognitive impairment, stroke, vascular dementia, cind, psd, nihss, iqcode, barthel index

## Abstract

Background: Vascular dementia is extremely common and contributes to stroke-associated morbidity and mortality. The study of vascular dementia may help to plan preventive interventions.

Aims: To study the frequency of cognitive impairment after stroke in a series of consecutive patients with acute stroke, along with factors which influence it.

Methods: Fifty adults with acute infarct or hemorrhage (as seen on computed tomography of the brain) were included in the study. The National Institute of Health Stroke Scale (NIHSS) and Barthel’s Index scores were done. Cognitive testing was done by PGI Battery of Brain Dysfunction (PGI-BBD) and Short Form of the Informant Questionnaire on Cognitive Decline in the Elderly (SIQCODE). Statistical analysis was by Student’s t-test, Chi-square test, Fisher’s exact test, and Mann-Whitney U test.

Results: Mean age of patients was 61.82 years; males and ischemic strokes predominated. Dementia was seen in 30%, cognitive impairment no dementia (CIND) in 42%, and normal cognition in 28% patients. Factors associated with vascular cognitive impairment included old age, male sex, low education, hemorrhages, recurrent or severe stroke, silent infarcts, severe cortical atrophy, and left hemispheric or subcortical involvement.

Conclusions: Up to 72% of patients have some form of cognitive impairment after a stroke. Secondary stroke prevention could reduce the incidence of vascular dementia.

## Introduction

Vascular dementia is one of the two most prevalent forms of dementia. Studies have demonstrated that post-stroke dementia (PSD) increases the risk for recurrent stroke and mortality [1]. Post-stroke dementia (PSD) is defined as the presence of dementia identified at three months after an acute, either recurrent or first-ever, stroke. A stroke increases the risk of dementia four to 12 times. The prevalence of PSD among stroke patients varies from 6% to 55% and may decline years after stroke [2]. Some patients may have cognitive impairment that does not meet the criteria for dementia but nevertheless have an increased risk of cognitive deterioration, institutionalization, and death. These patients may have more opportunities for treatment and prevention [3]. The study of predictors of dementia after a stroke may assist in planning interventions to prevent vascular dementia [4]. 

## Materials and methods

This was a prospective study of 50 consecutive patients of stroke attending the emergency or outpatient department. The Scientific Review Committee of Indraprastha Apollo Hospital approved this study. No approval number was required for this project. Patients included were those aged 45 years or above who had a stroke within one week of the first visit, irrespective of their previous cerebrovascular or cognitive status. Patients with transient ischemic attack, stroke associated with other brain lesions (e.g., tumour or trauma), other neurodegenerative disorders which may lead to dementia (e.g., Parkinsonism), and patients with persistent moderate to severe aphasia (score ≥ 1 on the language component of the National Institute of Health Stroke Scale, NIHSS) were excluded. Patients were evaluated on the first visit, at three months, and at 12 months. On the first visit, the diagnosis and type of stroke were established. Demographic details, handedness, education, past family and medication history, and risk factors for stroke were noted along with the BP on admission and any hypotensive episodes (BP < 105 systolic) during the hospital stay. Physical examination was done, including NIHSS scoring to assess stroke severity. A non-contrast computerised tomography (NCCT) of the head was performed to document the site and size of the infarct or haemorrhage and to look for strokes in strategic locations and silent infarcts [5].

The severity of white matter changes (WMC) was graded as 0, 1 or 2. In Grade 1, the abnormality was restricted to the region adjoining the ventricles. In Grade 2, the abnormality involved the entire region from lateral ventricle to the cortex. The grades given to the regions were added to give an overall value between 0 and 4 [6]. The measurements for cortical atrophy were taken from the CT slice that best depicted the third ventricle [7].

Atrophy was classified as absent if the maximal third ventricle width was less than 5 mm, mild if 5 to 6 mm, moderate if 6 to 7 mm, and severe if 7 mm and above. Pre-stroke cognitive status was assessed by means of the Short Form of the Informant Questionnaire on Cognitive Decline in the Elderly (Short IQCODE) and the functional status by the Barthel index. These were administered to the patient’s primary caregiver on all visits. The PGI battery of brain dysfunction (PGI BBD) was applied to all patients at three and 12 months after stroke. This is a battery of neuropsychological tests specifically designed for Indian patients keeping cultural factors in mind. Worsening in IQCODE scores between the first visit and the third-month examination would, therefore, imply deterioration mainly produced by the stroke. The third month was taken as a post-stroke baseline to assure stable cognition. Changes between three months and 12 months would reflect cognitive evolution after the acute phase of the stroke. Fresh vascular events were reassessed on the three and 12-month visits, and demographic, neurological, and functional data were compared among all patients.

Vascular dementia was defined as per the Diagnostic and Statistical Manual of Mental Disorders criteria. These were assessed by the IQCODE, neuropsychological test battery, and the activities of daily living on the Barthel index. PGI BBD scores less than 17 was taken as normal, 18 to 29 as cognitive impairment no dementia (CIND), and more than 30 as dementia [8-9]. The results were statistically analysed and an attempt was made to draw inferences about the presence of post-stroke cognitive impairment with and without dementia, factors influencing its development, whether it is more often cortical or subcortical, and the evolution of cognitive impairment over time.

## Results

**Figure 1:**
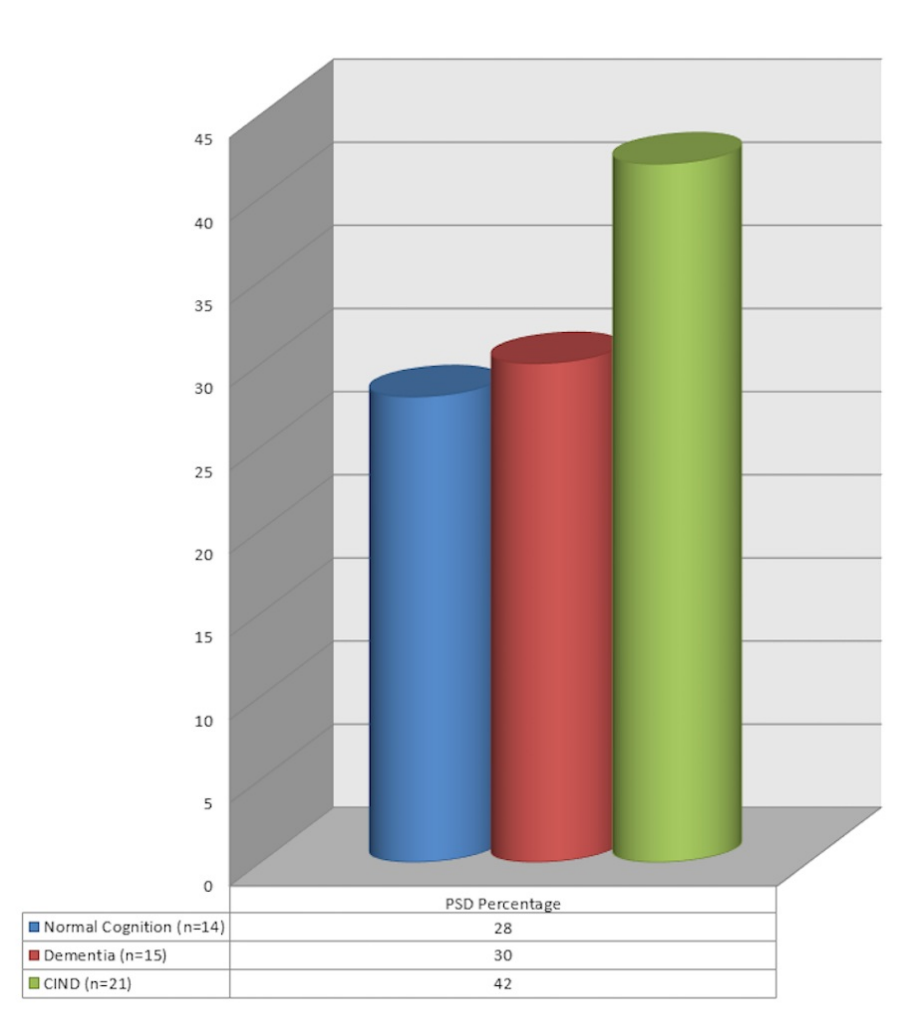
Post-Stroke Dementia

The study group comprised of 50 patients of whom 32 were males and 18 were females with a mean age of 61.82 years. Eighty percent had studied up to grade 10 or more. Infarcts were seen in 37 and haemorrhages in 13 patients. Of the 50 patients, 14 (28%) had normal cognition, 15 (30%) had dementia, and 21 (42%) had CIND as per the PGI BBD score (Figure 1). Thus, 36 (72%) had some form of cognitive impairment following a stroke. The domains that were commonly involved were retention for dissimilar pairs, visual retention, attention, and recognition.

The prevalence of cognitive impairment was higher with increasing age (100% in the age group 80 - 89 years), P = 0.695, and in male subjects (75% vs. 66.6% in females), P = 0.473. Of all the patients who studied until grade 10, 80% had cognitive impairment while only 70 % of all the patients who studied beyond grade 10 had cognitive impairment, P = 0.100. More patients with hemorrhagic strokes (84.6%) had cognitive impairment compared to those with ischemic strokes (67.5%), P = 0.077. Five of 50 patients had a recurrence of stroke, and of those patients, four (80%) had cognitive impairment (2 CIND, 2 dementia). Of the remaining 45 patients with no stroke recurrence, 32 (71.1%) had cognitive impairment (19 CIND, 13 dementia).

Of the 50 study patients, only one had CIND before stroke (SIQCODE 3.31). No relation of cognitive impairment was found with the level of glycemic control. Only four patients had hypotensive episodes in the post-stroke period, of whom two had CIND and two normal cognition. No particular vascular risk factor was associated with a greater risk of cognitive decline. Patients with dementia had a higher median NIHSS score of 6 (a greater disability at the time of stroke) and a lower median Barthel index of 12 (a greater dependency for activities of daily living). No obvious correlation was found with the size of the lesion or the degree of WMC on CT scan. The mean WMC score was 1.42 for patients with normal cognition, 1.42 for patients with CIND, and 1.26 for patients with dementia, P = 1.00.

**Figure 2:**
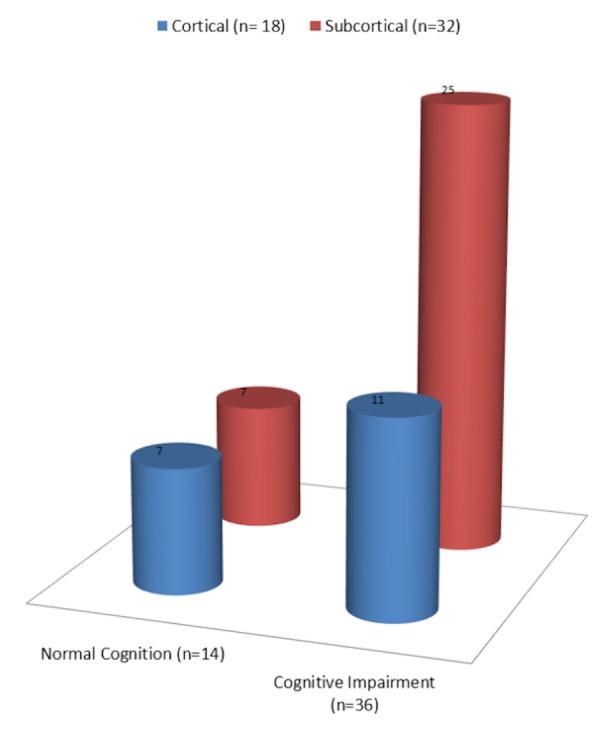
Cognitive impairment association with the location of lesion

Cognitive impairment was more frequent in patients with left-sided lesions (80.7%), P = 0.176, silent infarcts (88.8%), P = 0.694, and cortical atrophy (100% in severe atrophy), P = 0.337. Of the 36 patients with some form of cognitive impairment, the location of the lesion was subcortical in 25 and cortical in 11 subjects. Of the 14 patients with normal cognition, the location of the lesion was subcortical in seven and cortical in the other seven subjects (Figure 2). Of the 32 patients with subcortical distribution of the infarct or haemorrhage, 25 (78.1%) had some form of vascular cognitive impairment. Of the 18 patients with cortical distribution of the infarct or haemorrhage, 11 (61.1%) had some form of vascular cognitive impairment. There were 24 patients with lesions in strategic locations, 17 (70.8%) of whom had some form of cognitive impairment. Of the 26 patients with lesions in non-strategic locations, 19 (73%) had some form of cognitive impairment. Statistical tests applied were Student’s t-test, Mann-Whitney U test, Fisher’s exact test, and Chi-square test but none of the associations were statistically significant.

## Discussion

The distribution of infarcts and haemorrhages in this study reflected the usual pattern seen in stroke patients [2]. The mean age of the study subjects was 61.82 years, which was lower than that seen in most other studies [1, 10-12]. The mean age of Indian patients with stroke ranges from 63 - 65 years for men and 57 - 68 years for women. In this study, the ratio of men to women with stroke was 2:1. The male/female sex ratio for stroke patients in India is 7:1, which may be due to smoking and drinking being more prevalent among men than women [13]. These risk factors, however, are also common among urban, rich women, which may explain why strokes were common in women in this study.

Sundar, et al. studied 164 patients with stroke and concluded that 31.7% patients had cognitive impairment at three months post-ischemic stroke, either on the Mini-Mental State Examination (MMSE) or the frontal assessment battery (FAB) [14]. Of the study subjects, 17.07% were impaired on frontal executive functions only. Single and multiple infarcts were not significantly different with respect to post-stroke cognitive impairment, the main difference being that memory affection was significantly more common in the latter; hence, the importance of the need to be aware of disorders of these specialized functions, which directly impact the quality of life post-stroke, cannot be over-emphasized [14].

Das, et al. studied 960 patients and concluded that an overall prevalence of mild cognitive impairment (MCI) detected based on neuropsychological testing was 14.89% (95% CI: 12.19 to 17.95) [15]. Prevalence of the amnestic type was 6.04% (95% CI: 4.40 to 8.1) and that of the multiple domain type was 8.85% (95% CI: 6.81 to 11.32). Adjusted for age, education, and gender, the amnestic type was more common among men and the multiple domain types among women with the advancement of age. Rates differed considerably with educational attainment. Hypertension and diabetes mellitus were the major risk factors for both types of MCI [15].

Mukhopadhyay, et al. studied the prevalence of stroke and post-stroke cognitive impairment in the elderly and concluded that prevalence rates of stroke from their study were found to be comparable to rates from contemporary Indian studies and higher than older studies [16]. The rates are lower than those in more developed countries, which may be due to higher mortality in this particular low-income group. The continuous population ageing process in India, in conjunction with the increasing incidence of diabetes and hypertension, can cause an alarming rise in the incidence and prevalence of stroke, besides adversely affecting post-stroke residual disability and cognitive function. There is a pressing need for population-based awareness initiatives on healthy lifestyles. Existing treatment and rehabilitation facilities require upgrading, together with early diagnosis and adequate treatment of risk factors, especially for the underprivileged sections of society [16].

In the present study, vascular cognitive impairment was present in 36 (72%) of our patients, of whom 15 (30%) had dementia and 21 (42%) had CIND. The prevalence of PSD among recurrent or first-ever stroke patients varies from 6% to 55% [2, 10-12]. The difference of the prevalence of PSD among various studies depends on the study design (e.g., non-prospective or non-consecutive samples), the demographic characteristics of the population studied (e.g., age, gender, and ethnicity), criteria used for the diagnosis of dementia, the pre-existing cognitive level, lesion-related and radiological-associated factors (like exclusion of haemorrhage or recurrent stroke, white matter changes, and the presence of cerebral atrophy), vascular risk factors, the time interval between the stroke and the neuropsychological assessment, and length of follow-up [2].

Although our study group included more females and a relatively younger population as compared to other studies, the prevalence of dementia was not low. This may have been due to the fact that haemorrhages and recurrent strokes were also included in this study. Moreover, the incidence of  dementia in hospital-based studies like this one are much higher than the incidence reported from the community-based Framingham Study in which the rate of dementia was 19.3% in stroke patients compared to 11.0% in controls over a 10-year period [10]. This compares with an up to nine-fold increase reported in some cross-sectional studies. It is possible that strokes in the community sample were less severe. Cognitive dysfunction does not follow a linear time course after stroke. Short-term studies may over-diagnose cognitive post-stroke dysfunction. Further follow-up in this study may have revealed a decline in the prevalence of cognitive impairment [17]. The definition of vascular dementia used is an important consideration in the interpretation of prevalence rates. In our study, the presence of an infarct was not considered necessary for the diagnosis if the subject had extensive white matter pathology. This may have contributed to the high prevalence of vascular cognitive impairment.

In this study, the prevalence of cognitive impairment was higher with increasing age (100% in the age group 80 - 89 years) and in male subjects (75% vs. 66.6% in females). A lower level of education also appeared to be associated with a greater chance of cognitive impairment (80% in patients having studied till class 10 vs. 70% in those having studied further). More patients with hemorrhagic strokes (84.6%) had cognitive impairment compared to those with ischemic strokes (67.5%). There were no previous comparative studies that compared the prevalence of cognitive impairment in ischemic and hemorrhagic strokes. On the other hand, increasing age (> 60 years) and low education have consistently emerged as risk factors for cognitive impairment after stroke [2, 10]. This was considered to be due to the additional cerebrovascular pathology in older patients, which may be due to previous infarctions and non-infarct ischemic changes.

The role of Alzheimer’s disease (AD) type pathology in older patients is another important factor. Patients with higher educational attainment have a larger functional cognitive reserve and differences in lifestyle and risk factor profile, which are protective against cognitive decline. Although patients with stroke recurrence tended to have a higher chance of cognitive impairment (80% vs. 71% in those without stroke recurrence), no particular vascular risk factor was associated with a greater risk of cognitive decline, in our study group. Patients with cognitive impairment had a higher median NIHSS score (a greater disability at the time of stroke) and a lower median Barthel index (a greater dependency for activities of daily living), as in other studies. However, stroke recurrence did not predispose to cognitive impairment in all studies and neither did any particular risk factor, although cognitively impaired patients had a higher overall number of risk factors in comparison to non-impaired patients [10].

In our study, left-sided lesions (80.7%), the presence of silent infarcts (88.8%), and cortical atrophy (100% in severe atrophy) correlated with a greater risk of cognitive impairment but the size or volume of the lesion and degree of white matter change had no correlation. The strategic location of the lesion did not appear to be associated with a higher incidence of cognitive impairment either (70.8% in strategic lesions vs. 73% in non-strategic lesions). Other studies have found a correlation with size or volume and strategic location of the lesion [10]. The classic concept implies that dementia of vascular origin is the result of a critical volume of infarcted brain tissue. Large-sized infarctions are expected to produce more cognitive impairment compared to small-sized infarctions unless the small infarcts are in strategic locations (e.g., thalamus, internal capsule, basal ganglia, corpus callosum, and hippocampus) [18]. In other studies as well, left-sided strokes had a greater association with cognitive impairment even when patients with severe dysphasia were excluded. This is presumably because most memory tasks rely on intact language function. In most studies, no significant differences were found in the prevalence of dementia between patients with mild, moderate, and severe WMCs. The subcortical syndrome with executive deficits and mental slowing are the most prominent cognitive characteristics associated with severe WMCs, and these characteristics may lead to secondary impairments of memory and visuospatial functions [19]. Studies have shown that cortical atrophy predicts subsequent cognitive decline in both AD and PSD. Preventing strokes should thus be considered complementary to therapies for AD, and the two therapeutic strategies may produce synergistic effects [20].

In our study of the 36 patients with cognitive impairment, 25 (70%) cases had a subcortical location of the lesion. CIND was found more often than dementia in these patients. In contrast, the remaining patients with a cortical location of the lesion had an equal distribution of CIND and dementia. Patients with a subcortical location of a lesion were more likely to have cognitive impairment (78.1%) than those with a cortical location of a lesion (61.1%). Basal ganglia and thalamic lesions were especially associated with cognitive impairment. The cognitive profile of subcortical vascular disease can be distinguished from that of AD principally by milder memory impairment but more pronounced impairment of executive function and a slowing of motor and psychomotor speed. Because most studies have not included measures of motor speed in their test battery, the prevalence of subcortical dementia has been found to be low [18].

The main limitation of this study was that the number of subjects was small, and hence, none of the results achieved statistical significance. The results reflect the incidence in a predominantly urban population in a tertiary care hospital. Ischemic and hemorrhagic strokes should have been studied separately as the evolution of cognition may be different in both. The neuropsychological test battery did not effectively identify frontal lobe dysfunction or differentiate between cortical and subcortical dementia. MRI scans of the brain would have been a better imaging modality than CT to pick up radiological evidence of vascular brain damage. However, this was not done due to cost concerns. Further evolution of cognition over time and the effect of treatment could not be studied as no patients were available for follow-up. The strengths of the study were that it was a prospective study and one of the few such studies done in India. The neuropsychological test battery used was one that was validated in Indian patients and was performed by a single observer so there was no observer bias.

## Conclusions

In our study, we found that up to 72% of patients have some form of cognitive impairment after a stroke of which 30% had dementia and 42% had CIND. Neuropsychological assessment should be an important part of the clinical evaluation in stroke patients but is best done after about three months once the stroke has stabilised. Secondary stroke prevention could reduce the incidence of vascular dementia. This can be done by aiming at the modifiable risk factors for stroke, such as hypertension, diabetes, dyslipidemia, hyperhomocysteinemia, and smoking.
